# P-174. Burden, Epidemiology, and Clinical Characteristics of Leptospirosis within the Military Health System

**DOI:** 10.1093/ofid/ofae631.379

**Published:** 2025-01-29

**Authors:** Patrick J Graf, Xiuping Chu, Julian Davies, Patrick Hickey, Dana M Blyth

**Affiliations:** Walter Reed National Military Medical Center, Rockville, Maryland; Infectious Disease Clinical Research Program, The Henry M. Jackson Foundation for the Advancement of Military Medicine, Bethesda, Maryland; Infectious Diseases Clinical Research Program, Henry M. Jackson Foundation, Bethesda, Maryland; Uniformed Services University, Bethesda, Maryland; Walter Reed National Military Medical Center, Uniformed Services University, Bethesda, MD

## Abstract

**Background:**

Leptospirosis (Lepto) remains underreported due to diagnostic limitations. Military personnel are considered high risk due to deployments in endemic locations and exposure in contaminated training sites. We analyzed the frequency, epidemiology, and clinical characteristics of lepto diagnoses in the Military Health System (MHS).

**Figure 1:**
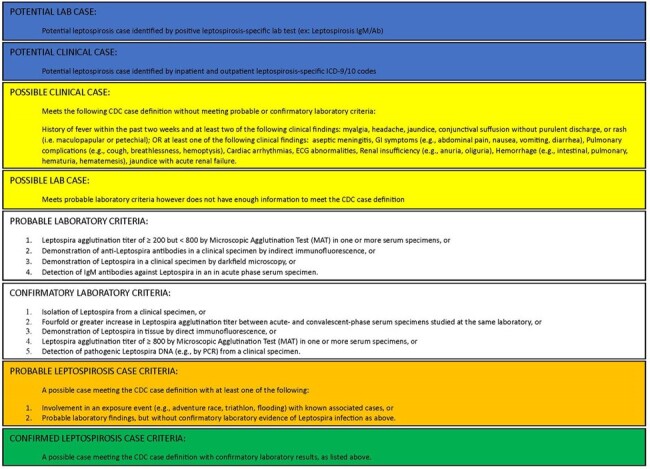
Leptospirosis Lab and Clinical Case Definitions and Criteria

**Methods:**

Potential MHS lepto diagnoses were identified by lepto-specific ICD-9/10 codes or positive lepto laboratory tests between 1/1/2013-1/1/2022. Potential cases underwent chart review, were classified per CDC case definitions (Figure 1), and compared for clinical and lab findings.

**Figure 2:**
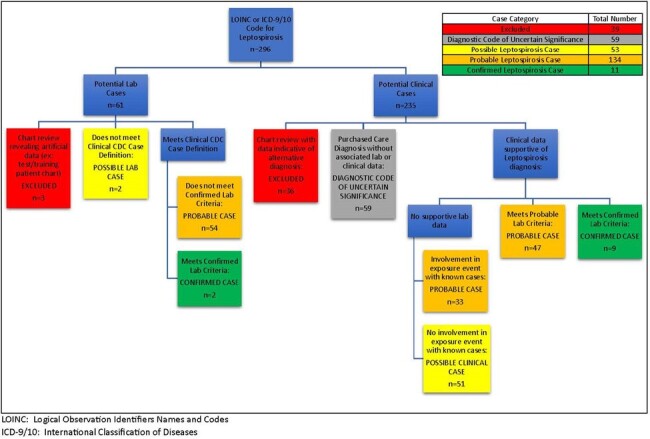
Flowchart for Identification of Leptospirosis Cases

**Results:**

Of 297 identified potential lepto cases, there were 11 confirmed, 134 probable, and 53 possible cases on chart review (Figure 2) of whom 77% (153/198) were hospitalized and one died (Table 1). Cases were confirmed via microscopic agglutination test (MAT, 6/11), blood culture (3/11), MAT and blood culture (1/11), and urine PCR (1/11) (Table 2). Of cases with demographic data, 89% were male (176/197) and 82% (162/197) were service members, with 16% (31/197) retired, dependents, or civilians. Of those with documented exposures, 52% (88/169) were recreational, and an additional 5 cases non-occupational (cumulatively 55%, 93/169). Fresh water exposures were common and more likely in confirmed/probable cases, with 67 associated with jungle warfare training in Okinawa. Of note, 44% of all cases (87/198) were diagnosed in those living in endemic areas without occupational exposure or deployment/travel. Japan (86/198), Guam (55/198), and Hawaii (22/198) were the most common areas of exposure. Confirmed cases were most likely to be exposed and diagnosed in Hawaii.

**Table 1:**
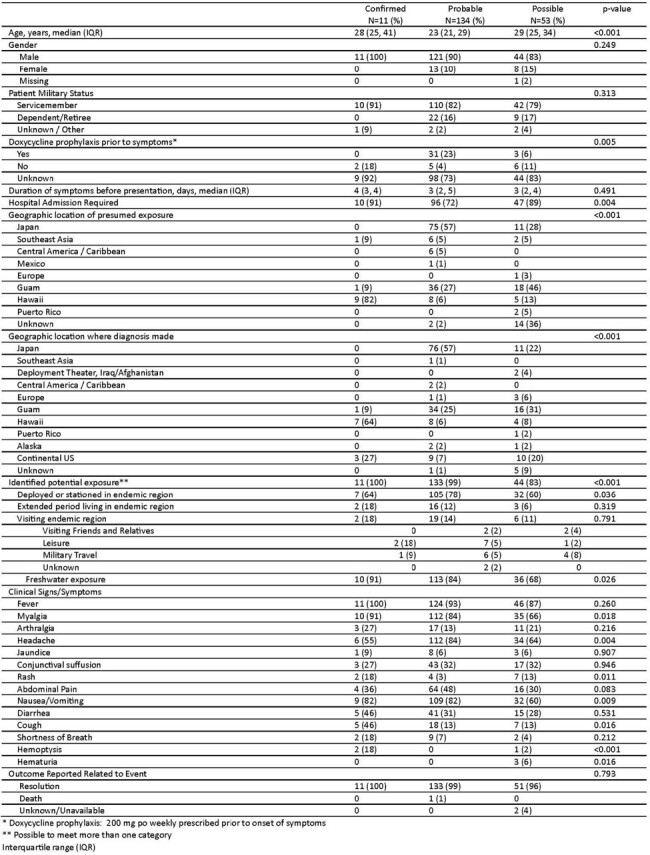
Clinical Characteristics of Confirmed, Probable, and Possible Leptospirosis Cases

**Conclusion:**

Lepto remains a common diagnosis within the MHS, especially among service members and in endemic areas. Despite classic teaching, a significant proportion of MHS cases occur in patients living in endemic areas without occupational or deployment exposures. This highlights lepto as a continual threat and burden on systems in endemic areas in addition to the classically recognized military exposures. Furthermore, despite a substantial amount of possible and probable cases, there remain few confirmed cases.

**Table 2:**
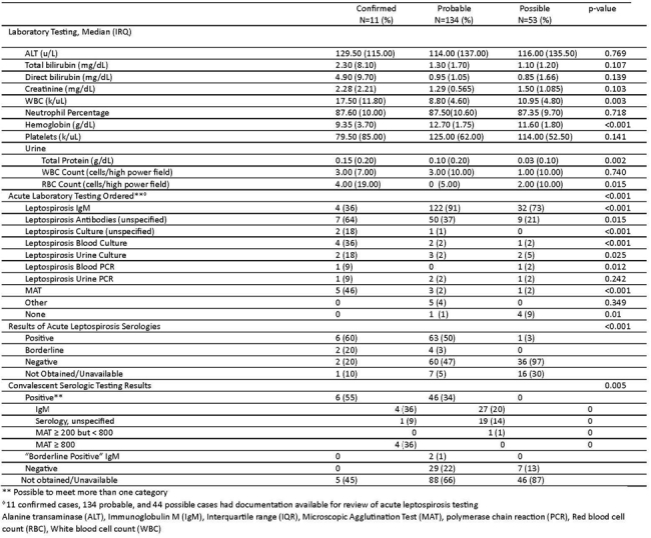
Laboratory Testing of Confirmed, Probable, and Possible Leptospirosis

**Disclosures:**

**All Authors**: No reported disclosures

